# ST2825, a Small Molecule Inhibitor of MyD88, Suppresses NF-κB Activation and the ROS/NLRP3/Cleaved Caspase-1 Signaling Pathway to Attenuate Lipopolysaccharide-Stimulated Neuroinflammation

**DOI:** 10.3390/molecules27092990

**Published:** 2022-05-06

**Authors:** Shan-Shan Zhang, Man Liu, Dong-Ni Liu, Yu-Fu Shang, Yue-Hua Wang, Guan-Hua Du

**Affiliations:** 1 State Key Laboratory of Bioactive Substance and Function of Natural Medicines, Institute of Materia Medica, Chinese Academy of Medical Sciences & Peking Union Medical College, Beijing 100050, China; zhangshanshan@imm.ac.cn (S.-S.Z.); liuman@imm.ac.cn (M.L.); liudongni@imm.ac.cn (D.-N.L.); shangyufu@imm.ac.cn (Y.-F.S.); 2 Beijing Key Laboratory of Drug Target Identification and New Drug Screening, Institute of Materia Medica, Chinese Academy of Medical Sciences & Peking Union Medical College, Beijing 100050, China

**Keywords:** neuroinflammation, ST2825, BV2 microglia cells, lipopolysaccharide (LPS)

## Abstract

Neuroinflammation characterized by microglia activation is the mechanism of the occurrence and development of various central nervous system diseases. ST2825, as a peptide-mimetic MyD88 homodimerization inhibitor, has been identified as crucial molecule with an anti-inflammatory role in several immune cells, especially microglia. The purpose of the study was to investigate the anti-neuroinflammatory effects and the possible mechanism of ST2825. **Methods**: Lipopolysaccharide (LPS) was used to stimulate neuroinflammation in male BALB/c mice and BV2 microglia cells. The NO level was determined by Griess Reagents. The levels of pro-inflammatory cytokines and chemokines were determined by ELISA. The expressions of inflammatory proteins were determined by real-time PCR and Western blotting analysis. The level of ROS was detected by DCFH-DA staining. **Results**: In vivo, the improved levels of LPS-induced pro-inflammatory factors, including TNF-α, IL-6, IL-1β, MCP-1 and ICAM-1 in the cortex and hippocampus, were reduced after ST2825 treatment. In vitro, the levels of LPS-induced pro-inflammatory factors, including NO, TNF-α, IL-6, IL-1β, MCP-1, iNOS, COX2 and ROS, were remarkably decreased after ST2825 treatment. Further research found that the mechanism of its anti-neuroinflammatory effects appeared to be associated with inhibition of NF-κB activation and down-regulation of the NLRP3/cleaved caspase-1 signaling pathway. **Conclusions**: The current findings provide new insights into the activity and molecular mechanism of ST2825 for the treatment of neuroinflammation.

## 1. Introduction

Neuroinflammation is characterized by microglia activation, which is closely related to the pathogenesis of neurodegenerative diseases such as Alzheimer’s disease, Parkinson’s disease, Multiple sclerosis, Huntington’s disease, etc. Microglia are the resident immune cells of the central nervous system, which play an important role in maintaining tissue homeostasis under normal conditions. However, when there is a neuronal injury or other insult, microglia will be activated to secrete pro-inflammatory factors. Excessive microglial activation damages the surrounding healthy neural tissue [1,2,3]. In response to brain tissue damage, activated microglia release a high concentration of NO which, as a chemoattractant, recruits more microglia to migrate to the injury site, promotes its chronic activation and contributes to long-term neurodegeneration [3,4].

In general, microglia express numerous pattern recognition receptors such as the Toll-like receptor (TLR) 4, which can be activated by LPS, a vital molecular component of the cell outer membrane of gram-negative bacteria, followed by the activation of NF-kB and inflammatory cascades [5,6,7,8]. The Myeloid differentiation primary response protein 88 (MyD88) is an adaptor protein for the TLR4 and interleukin-1 receptor (IL-1R) families, leading to cytokine release, transformation of cell phenotype and migration of microglia and macrophages to injury sites [9]. Due to this fact, the identification of new molecules that regulate the TLR4/MyD88 signaling pathway has become a priority.

**Figure 1:**
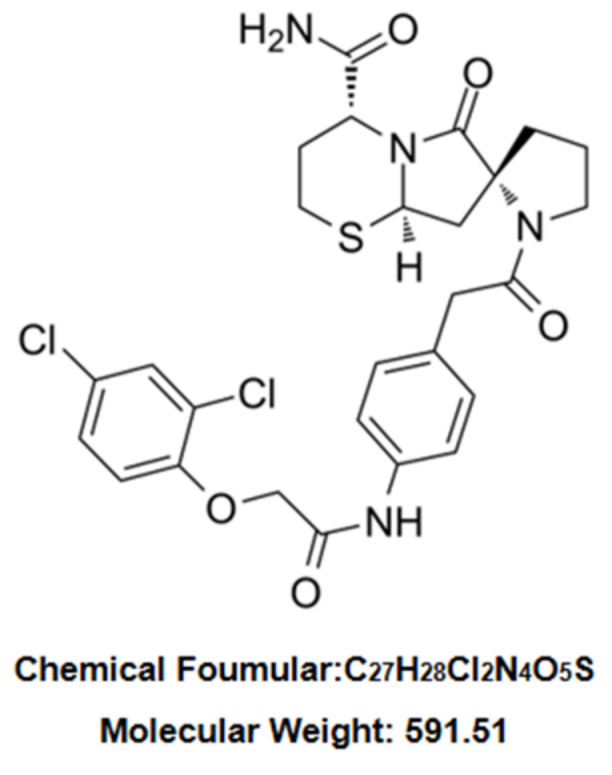
Chemical structure of ST2825.

ST2825 (Figure 1), a synthetic analogue of MyD88, is a MyD88-specific inhibitor, by virtue of interfering with MyD88 homodimerization. ST2825 has been applied in different models of human diseases including lymphoma, leukemia, human hepatocellular carcinoma and traumatic brain injury [6]. In particular, the anti-inflammatory effects of ST2825 have been demonstrated through the inhibition of various pro-inflammatory cytokines in PBMC [6] and RAW264.7 cells [10] stimulated by LPS. Given these findings, the purpose of this study was to investigate the effects of ST2825 against LPS-induced inflammation, and to analyze the possible molecular mechanism involved.

## 2. Results

### 2.1. ST2825 Reduced Pro-Inflammatory Factors Production in LPS-Stimulated Mice

**Figure 2:**
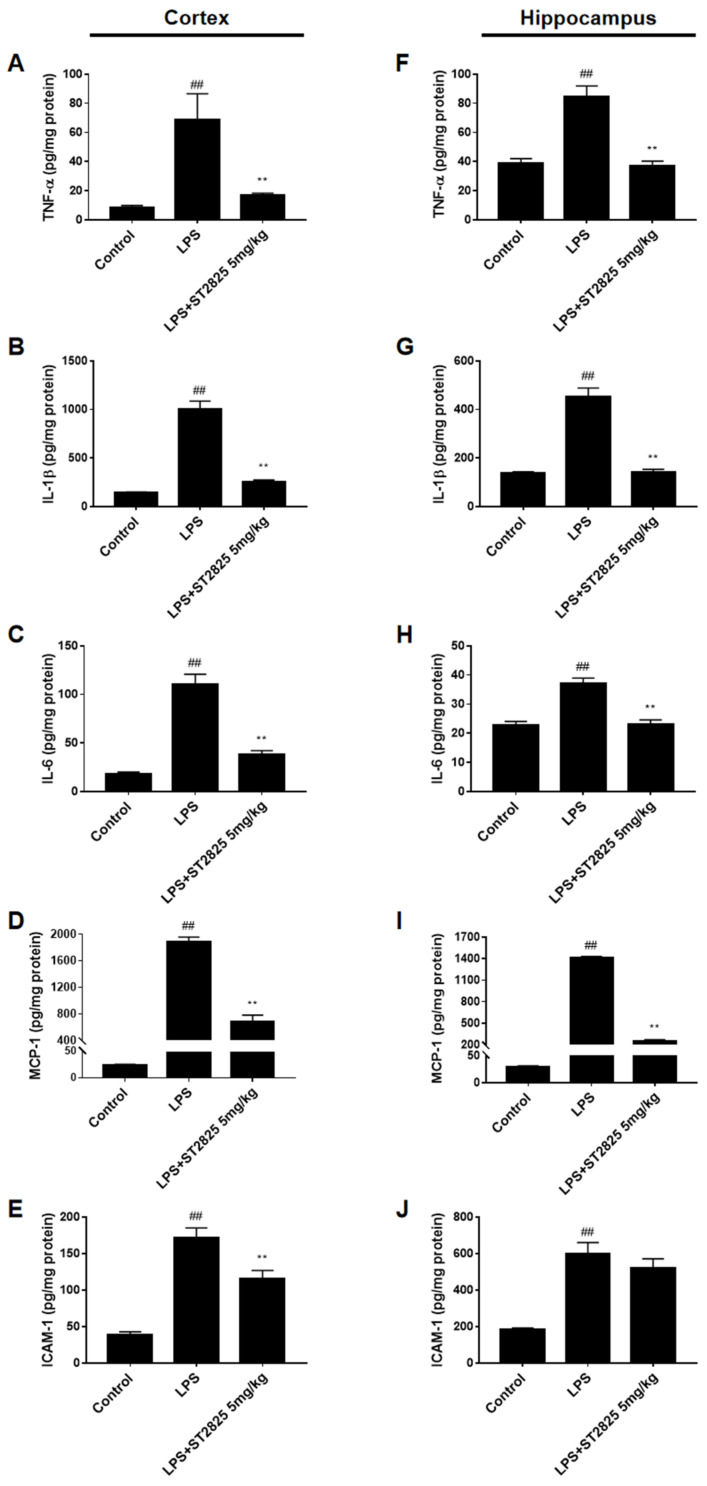
Effects of ST2825 on LPS-induced pro-inflammatory factors in BALB/c mice. The levels of (**A**) TNF-α, (**B**) IL-1β, (**C**) IL-6, (**D**) MCP-1, (**E**) ICAM-1 in the cortex and (**F**) TNF-α, (**G**) IL-1β, (**H**) IL-6, (**I**) MCP-1, (**J**) ICAM-1 in the hippocampus were assessed by ELISA. Values are mean ± SEM (*n* = 6). ## *p* < 0.01 vs. control group; ** *p* < 0.01 vs. LPS group.

In order to investigate whether ST2825 has an anti-inflammatory effect in vivo, we detected the levels of cytokines (such as TNF-α, IL-1β and IL-6), chemokine (such as MCP-1), and adhesion molecules (such as ICAM-1), in the cerebral cortex and hippocampus of BALB/c mice stimulated by LPS. Based on our previous experience in LPS-stimulated mice models [7], 5 mg/kg of ST2825 was selected for administration. For the administration group, mice were treated with ST2825 6 h before LPS stimulation, taking into consideration that ST2825 is a specific inhibitor of the MyD88 TIR domain. Our data revealed that, compared with the control group, these pro-inflammatory factors—especially IL-1β and MCP-1 (*p* < 0.01)—were significantly higher in the cortex and hippocampus of the LPS model group, and that ST2825 apparently reduced the levels of these inflammatory factors, except for ICAM-1 in the hippocampus (*p* < 0.01, Figure 2).

### 2.2. ST2825 Decreased Pro-Inflammatory Factors and Increased Anti-Inflammatory Factors in LPS-Stimulated BV2 Cells

**Figure 3:**
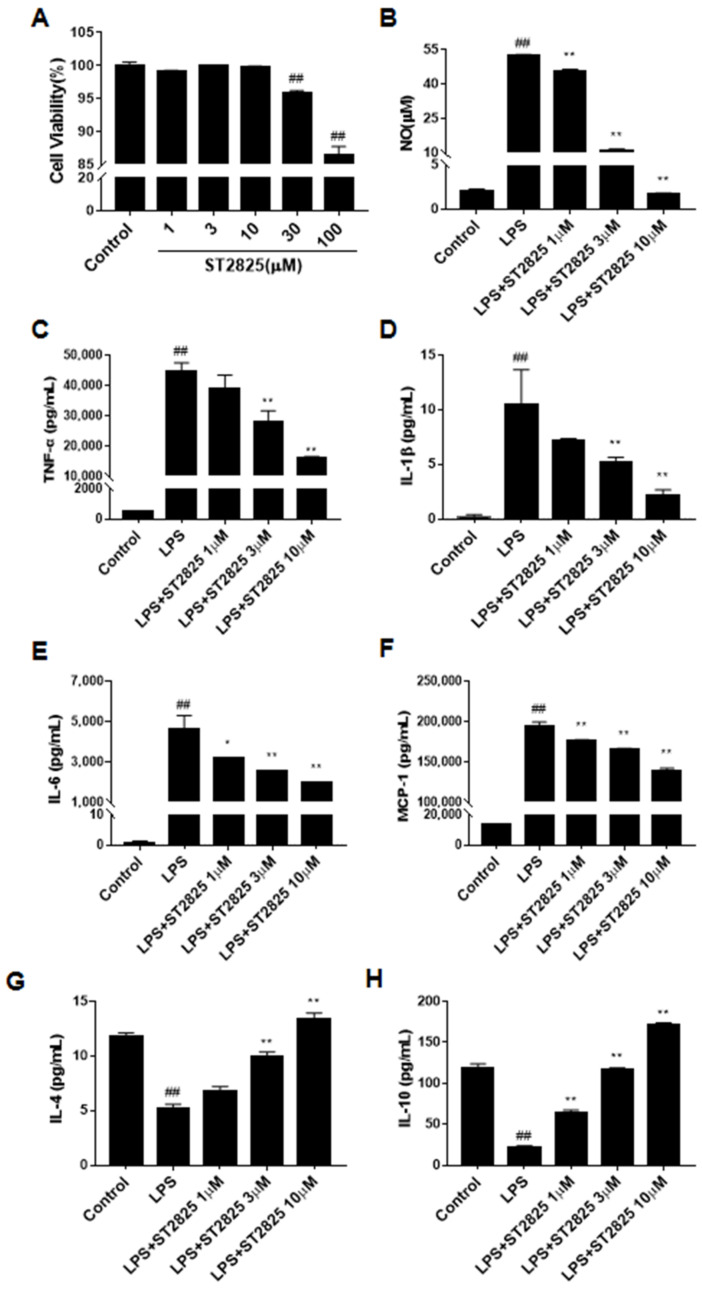
Effects of ST2825 on LPS-induced pro-inflammatory factors in BV2 cells. (**A**) Cell viability of ST2825 on BV2 microglia cells. (**B**) Effect of ST2825 on NO production. The levels of (**C**) TNF-α, (**D**) IL-1β, (**E**) IL-6, (**F**) MCP-1, (**G**) IL-4 and (**H**) IL-10 in the culture supernatant were assessed by ELISA. Values are mean ± SEM (*n =* 3). ## *p* < 0.01 vs. control group; * *p* < 0.05, ** *p* < 0.01 vs. LPS group.

The CCK-8 assay showed that ST2825 itself had no obvious cytotoxicity at 1, 3 and 10 µM in the BV2 microglial cells after 24 h treatment (Figure 3A), therefore we chose concentrations at 10 μM or lower for all subsequent experiments. As an initial indicator of the anti-neuroinflammatory effects of ST2825 on LPS-stimulated BV2 cells, the concentration of NO was assessed. The results showed that ST2825 pretreatment markedly decreased the level of NO (*p <* 0.01, Figure 3B). In order to further explore the anti-neuroinflammatory effects of ST2825 on LPS-stimulated BV2 cells, we detected the levels of inflammatory cytokines, including TNF-α, IL-1β, IL-6 and chemokine MCP-1. As shown in Figure 3C–F, compared with the control group, the levels of these pro-inflammatory factors in the LPS model group increased significantly (*p <* 0.01), while ST2825 pretreatment observably decreased their levels (*p <* 0.01). The above results suggested that ST2825 inhibited the production of these pro-inflammatory factors. As shown in Figure 3G,H, compared with the control group, the levels of anti-inflammatory factors in the LPS model group decreased significantly (*p <* 0.01), while ST2825 pretreatment observably increased their levels (*p <* 0.01) in a dose-dependent manner, which suggested that ST2825 promotes the production of these anti-inflammatory factors.

### 2.3. ST2825 Decreased the Expression of iNOS and COX-2 in LPS-Stimulated BV2 Cells 

**Figure 4:**
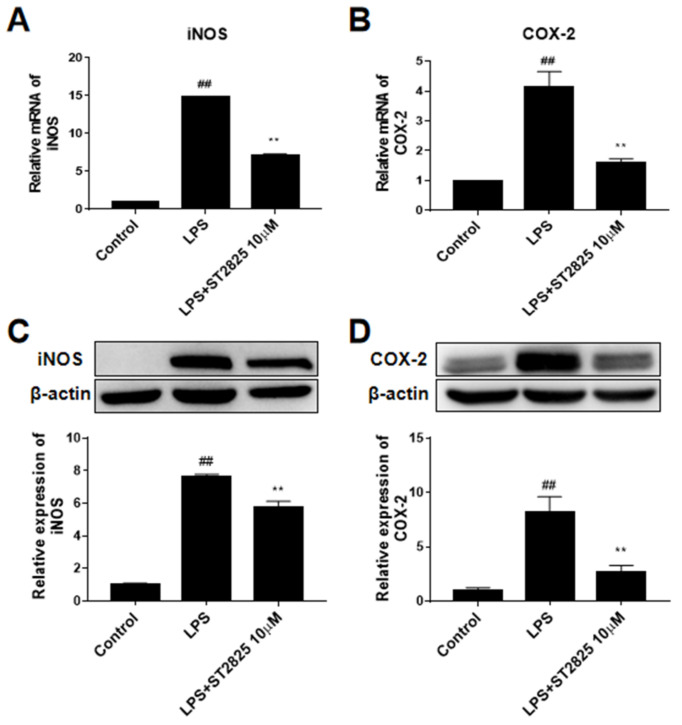
Effects of ST2825 on the production of LPS-induced iNOS and COX-2 in BV2 cells. Relative mRNA levels of (**A**) iNOS and (**B**) COX-2 were assessed by RT-PCR. The protein levels of (**C**) iNOS and (**D**) COX-2 were assessed by Western blot. Values are mean ± SEM (*n =* 3). ## *p* < 0.01 vs. control group; ** *p* < 0.01 vs. LPS group.

As shown in Figure 4, the mRNA levels of iNOS and COX-2 in the LPS model group increased in varying degrees after LPS stimulation for 6 h (*p <* 0.01), and ST2825 pretreatment significantly decreased the transcriptional levels of iNOS (*p <* 0.01, Figure 4A) and COX-2 (*p <* 0.01, Figure 4B). This observation led us to further investigate the effect of ST2825 on the protein expression of iNOS and COX-2 in LPS-treated BV2 cells. The results showed that, compared with the control group, the protein expression levels of iNOS and COX-2 in the LPS model group increased to about eight times (*p <* 0.01), while ST2825 pretreatment observably decreased the protein expression of iNOS (*p <* 0.01, Figure 4C) and COX-2 (*p <* 0.01, Figure 4D).

### 2.4. ST2825 Inhibited NF-κB Activation and Down-Regulated NLRP3/Cleaved Caspase-1 Signaling Pathway in LPS-Stimulated BV2 Cells

**Figure 5:**
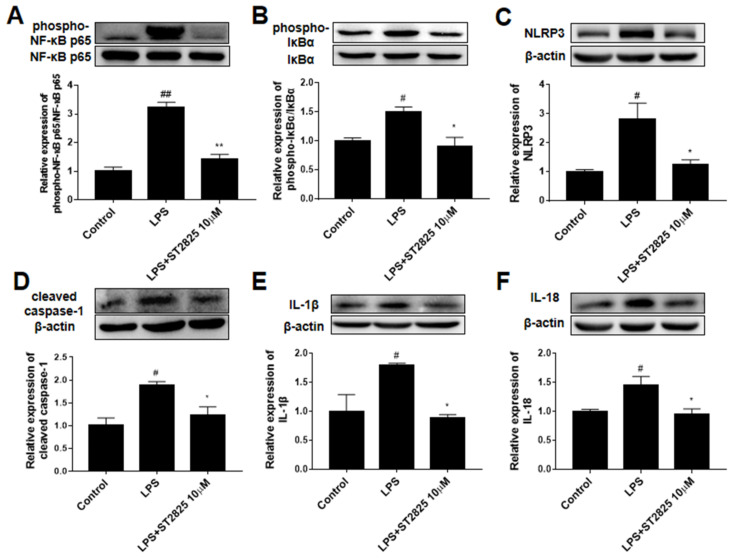
Effects of ST2825 on LPS-induced NF-κB activation and the NLRP3/cleaved caspase-1 signaling pathway in BV2 cells. The protein levels of (**A**) phospho-NF-κB p65, (**B**) phospho-IκBα, (**C**) NLRP3, (**D**) cleaved caspase-1, (**E**) IL-1β, (**F**) IL-18. Values are mean ± SEM (*n =* 3). # *p* < 0.05, ## *p* < 0.01 vs. control group; * *p* < 0.05, ** *p* < 0.01 vs. LPS group.

To elucidate the potential mechanism underlying the anti-inflammatory effects of ST2825, its effects on NF-κB activation and the NLRP3/cleaved caspase-1 signaling pathway were investigated by Western blot analysis. As shown in Figure 5, compared with the control group, the protein phosphorylation level of NF-κB (*p <* 0.01, Figure 5A) and IκBα (*p <* 0.05, Figure 5B) as well as the protein expression levels of NLRP3 (*p <* 0.05, Figure 5C), cleaved caspase-1 (*p <* 0.05, Figure 5D), IL-1 (*p <* 0.05, Figure 5E) and IL-18 (*p <* 0.05, Figure 5F) in the LPS group were significantly increased. ST2825 pretreatment significantly inhibited the expression level of NLRP3 (*p <* 0.05, Figure 5C), cleaved caspase-1 (*p <* 0.05, Figure 5D), IL-1β (*p <* 0.05, Figure 5E) and IL-18 (*p <* 0.05, Figure 5F) as well the activation of NF-κB (*p <* 0.01, Figure 5A) and IκBα (*p <* 0.05, Figure 5B). These results showed that ST2825 inhibited the activation of NF-κB and the expression of the NLRP3/ cleaved caspase-1 signaling pathway.

### 2.5. ST2825 Inhibited ROS Accumulation in LPS-Stimulated BV2 Cells

**Figure 6:**
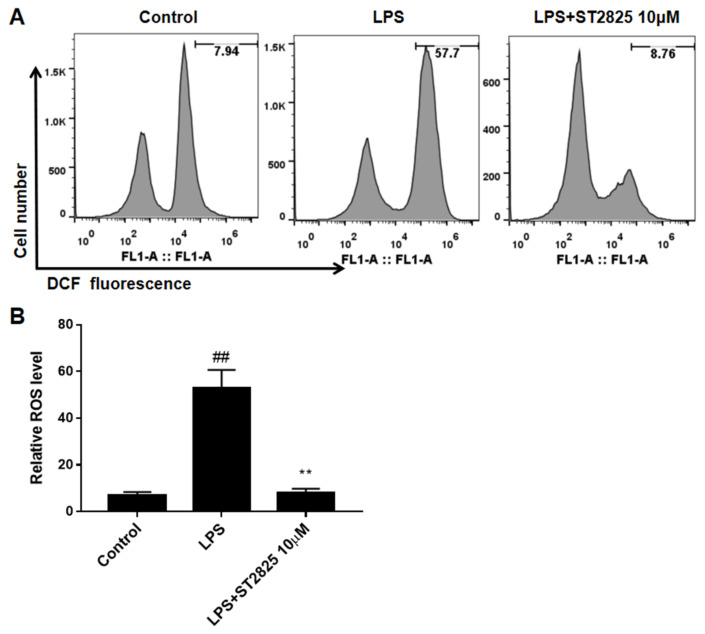
Effects of ST2825 on LPS-induced ROS production in BV2 cells. (**A**) ROS production measured by flow cytometry. (**B**) Quantitative analysis of DCFH-DA accumulation by FACS. Values are mean ± SEM (*n =* 3). ## *p* < 0.01 vs. control group; ** *p* < 0.01 vs. LPS group.

The inhibitory effect of ST2825 on the production of ROS was also analyzed by flow cytometry. As shown in Figure 6, the level of ROS increased after LPS stimulation (*p <* 0.01), while in ST2825-pretreated BV2 cells, the production of LPS-stimulated ROS was almost completely blocked (*p <* 0.01). These findings indicated that ST2825 had a strong ROS-scavenging effect.

## 3. Discussion

The central nervous system (CNS) is populated by neurons and glial cells, in which microglia account for 10–15% of the total number of adult brain glial cells [11]. Microglia play an important role in the regulation of innate immunity and neuroinflammation, and actively regulate the CNS development and maintain homeostasis [12]. However, under pathological conditions, neuronal cell death leads to microglia abnormal activation and the production of excessive neurotoxic factors, including NO, prostaglandin E2 ($PGE_{2}$), ROS and pro-inflammatory cytokines such as TNF-a, IL-1β and IL-6, which in turn leads to the gradual loss of neurons and the aggravation of inflammatory response [2,11]. Therefore, intervention in the activation process of microglia may be a promising method for the treatment of various CNS diseases.

Pathogens such as lipopolysaccharide (LPS) of Gram-negative bacteria, the peptidoglycan (PGN) of Gram-positive bacteria, β-amyloid, bacteria, and viruses can cause neuroinflammation, which is arguably the most destructive component of neurodegenerative processes initiated in part through the TLR4 signaling pathway activation [2,13,14]. LPS injection is a common method of performing an inflammatory response model, and is well-reproduced [7]. Upon stimulation with LPS, two classic signaling pathways are activated: MyD88-dependent and MyD88-independent pathways [15]. MyD88 is an adaptor protein common to all TLR signaling pathways, such that it forms a receptor complex—except TLR3 [16,17]—while the Toll/IL-1R (TIR) domain-containing adaptor-inducing IFN-β (TRIF) is solely engaged by TLR3 and TLR4, which mediate the induction of type 1 interferon-inducible genes [18,19].

**Figure 7:**
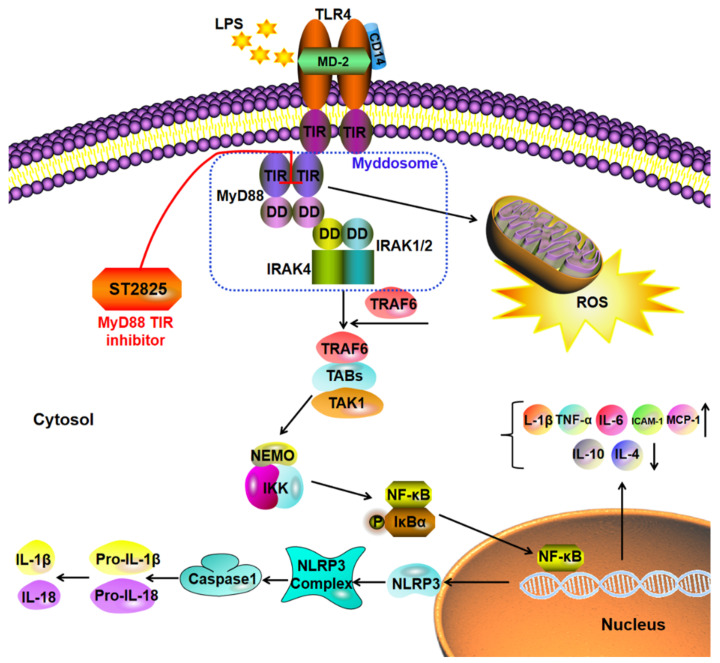
Schematic diagram of the potential anti-neuroinflammation molecular mechanism of ST2825 through NF-κB inhibition and the ROS/NLRP3/cleaved caspase-1 signaling pathway.

MyD88 consists of an N-terminal dead domain (DD) and a C-terminal TIR domain, separated by a short linker region [20]. It plays an important role in the pathogenesis of Alzheimer’s disease, Parkinson’s disease, amyotrophic lateral sclerosis, multiple system atrophy and related disorders [21]. MyD88 knockout mice lack the ability to respond to LPS [22]. The functional core of the MyD88 molecule is the ability of its TIR domain to heterodimerize with the TIR domain-containing adaptor protein (TIRAP) [23], and to homodimerize with another MyD88 molecule in the TIR domain [20,24]. By interacting with the IL-1R associated kinase (IRAK)-4 through its dead domain, MyD88 activates other members of the IRAK family, such as IRAK-4 and subsequently IRAK-1. The above process leads to the activation of the tumor necrosis factor (TNF), the receptor-associated factor 6 (TRAF6) and the E2 ubiquitin protein ligase, which activates the TAK1/TAK1 binding protein (TAB) complex to trigger MAPK and the NF-κB signaling pathway [25]. Mechanistically, ST2825 blocks MyD88-dependent signaling by binding to the BB ring and specifically interfering with MyD88 homodimerization of the TIR domains rather than homodimerization of the death domains [26,27], resulting in the reduction of Myddsome formation and pro-inflammatory cytokines production [20] (Figure 7).

The Cytokine and chemokine families mediate both immune cell recruitment and the complex intracellular signaling control mechanisms that characterize inflammation [28]. Cytokines including TNF-α, IL-1β and IL-6, participate in acute and chronic inflammation via signaling the type I cytokine receptor. It is generally believed that pro-inflammatory cytokines in the brain are synthesized in CNS after stimulation by peripheral LPS treatment, rather than cytokines released from the periphery being diffused through the blood-brain barrier [29]. Chemokines and their receptors are able to control the migration and residence of all immune cells [30]. As a typical chemokine, MCP-1 (CCL2) stimulates chemotaxis of monocytes, cytokine expression and ROS generation induced by respiratory burst [30]. ICAM-1, an adhesion molecule, regulates leukocyte rolling and adhesion, guides leukocyte crossing of the endothelial layer and promotes the regression of inflammatory response [31]. Our results showed that LPS induced the release of pro-inflammatory factors including TNF-α, IL-1β, IL-6, MCP-1 and ICAM-1 in the cortex and hippocampus of LPS-induced mice, while ST2825 pretreatment apparently reduced the levels of these inflammatory factors, except ICAM-1 in the hippocampus tissue. The reason may have been that the regulation of ICAM-1 in the hippocampus by ST2825 required a higher dose or longer duration of action. Similarly, the levels of pro-inflammatory factors TNF-α, IL-1β, IL-6 and MCP-1 were increased in the LPS-stimulated BV2 cells, while ST2825 pretreatment significantly decreased these pro-inflammatory factors in a dose-dependent manner. NO is produced when iNOS catalyzes the conversion of L-arginine to L-citrulline, and is another way in which neuroinflammation can directly influence neuronal apoptosis [32]. Excessive NO will lead to the production of pro-inflammatory cytokines, and then aggravate the inflammatory response and physiological dysfunction [33]. COX-2 has been identified as a key enzyme involved in inflammatory response due to its ability to catalyze the production of a variety of prostaglandins [3]. In this study, our data demonstrated that ST2825 pretreatment remarkably decreased LPS-induced NO release in a dose-dependent manner, as well as restraining the transcription and expression levels of iNOS and COX-2 protein induced by LPS. Based on the above results, we hold that ST2825 exhibited prominent anti-inflammatory effects on LPS-stimulated mice and BV2 cells.

The expression levels of inflammatory cytokines, including TNF-α and IL-6, are regulated by various transcription factors, one of the most important of which is NF-κB [15]. NF-κB is a heterologous trimer consisting of p50, p65, and IκBα subunits, and is normally inactive in the cytoplasm. It has been reported that ST2825 prevents subarachnoid hemorrhage-induced activation of the NF-κB signaling pathway [25]. Nevertheless, when cells are stimulated, IκB is phosphorylated, ubiquitinated and degraded, releasing NF-κB into the nucleus and inducing the transcription of pro-inflammatory cytokines. In the present study, ST2825 prominently inhibited the activation of NF-κB and IκBα in LPS-stimulated BV2 microglia. 

Inflammasomes, cytosolic multiprotein complexes, are responsible for the cleavage of caspase-1-mediated pro-inflammatory cytokines, such as IL-1β and IL-18 [33]. NLRP3 inflammasome is composed of NLRP3, ASC and caspase-1, and is one of the most typical inflammasomes. It is well-established that the activation of the TLR4/MyD88/NF-κB signaling pathway makes a great contribution to the activation of NLRP3 inflammasome. In this study, compared with the control group, the expression of NLRP3 and cleaved caspases-1 proteins increased significantly after LPS stimulation, indicating that the NLRP3/cleaved caspase-1 signaling pathway was activated after LPS stimulation. However, ST2825 pretreatment observably inhibited the LPS-induced up-regulation of NLRP3, cleaved caspase-1, mature IL-18 and IL-1β proteins, which may in turn break the IL-1β/MyD88/NF-κB/IL-1β cycle.

Oxidative stress, caused by a decreased anti-oxidant system and increased production of ROS, plays a vital role in the development of various degenerative diseases [3]. As a secondary messenger, intracellular ROS promotes inflammation by upregulating kinase cascades, which has attracted extensive attention [3]. ROS regulates the activation of NLRP3 inflammasome, promotes the release of various pro-inflammatory cytokines and accelerates the process of inflammatory response by altering kinase cascades and activating transcription factors, including MAPK and NF-κB [34]. After LPS stimulation, ROS accumulation in BV2 cells was greatly increased, while pretreatment with ST2825 almost completely counteracted LPS-induced ROS production, which proved that ST2825 exhibited a strong antioxidant effect.

## 4. Materials and Methods

### 4.1. Regents

ST2825 (Cat# HY-50937) was purchased from MedChemExpress (MCE, Shanghai, China). LPS (*Escherichia coli* 0127: B8) was obtained from Sigma–Aldrich (St. Louis, MO, USA). Dulbecco’s Modified Eagle Medium (DMEM) and fetal bovine serum (FBS) were purchased from Gibco BRL (Grand Island, NY, USA). Enzyme-lLinked immunos orbent assay (ELISA) kits of TNF-α (EM008), IL-1β (EM001), IL-6 (EM004), MCP-1 (EM018) and ICAM-1 (EM013) were purchased from ExCell Biology (Shanghai, China). Antibodies against iNOS, phospho-NF-κB p65, NF-κB p65, phospho-IκBα, IκBα, NLRP3, cleaved caspase-1 and β-actin were obtained from Cell Signaling Technology (Beverley, CA, USA). Antibodies against IL-1β, IL-18 and COX-2 were purchased from Abcam (Cambridge, UK).

### 4.2. Animals and Treatment

All animal care and animal experiment procedures were approved by the ethics committees of the Institute of Materia Medica, and the Chinese Academy of Medical Sciences & Peking Union Medical College. A total of 18 male BALB/c mice (weighing 18–22 g, 6–8 weeks old) were included in the experiment, and they were obtained from the Laboratory Animal Centre of Beijing Hua-Fu-Kang Bioscience Co., Ltd., (Beijing, China; the animal certification number was SCXK (Jing) 2014-0004). All animals were kept under a 12-h light/dark cycle, with lights with controlled temperature at 23 ± 2 °C and controlled humidity at 55% ± 5%. Three days after acclimation, the mice were divided into three groups: the control group (i.g. administration of saline); the LPS group (i.g. administration of saline 6 h before LPS injection and i.p. LPS 5 mg/kg) and the LPS + ST2825 5 mg/kg group (i.g. administration of ST2825 5 mg/kg 6 h before LPS injection and i.p. LPS 5 mg/kg) according to random number table, with 6 mice in each group. After 6 h of LPS stimulation, the mice were euthanized under 5% isoflurane anesthesia and decapitated according to the order of LPS stimulation. The cerebral cortex and hippocampus of the mice were isolated on ice and immediately frozen at −80 ℃ for further study [7,35,36].

### 4.3. BV2 Microglia Cells Culture and Treatment

BV2 microglia cells were maintained in DMED supplemented with 10% heat-inactivated FBS at 37 ℃ in a humidified incubator under 5% CO_2_. The BV2 cells were seeded into 96-well or 6-well plates, and pre-treated with a series of indicated concentrations of ST2825 (1, 3 and 10 μM) for 12 h before being incubated with or without 200 ng/mL LPS for another 24 h or 6 h. The ST2825 was dissolved with DMSO and diluted to the corresponding concentration with fresh DMEM. The final DMSO concentration was <0.05% in all experiments [37].

### 4.4. Cell Viability Assay

To determine the cell viability, BV2 cells were seeded into 96-well plates at a density of 2.5 × 10^4^ cells per well, and treated with different concentrations of ST2825 (1, 3, and 10 μM) for 24 h. Afterwards, the medium was removed and 10 μL CCK-8 (Dojindo Molecular Technologies, Inc., Kumamoto Techno Research Park, Kumamoto, Japan.) was added to each well. After incubation at 37 °C for 2 h, the optical density was measured at a wavelength of 450 nm using a microplate reader [37].

### 4.5. NO Assay

The BV2 cells were treated with different concentrations of ST2825 for 12 h, then stimulated with 200 ng/mL LPS for 24 h, and the culture supernatants were collected. The nitrite concentration in the medium, an indicator of NO production, was measured with the Griess reaction using NO analysis kit (Applygen Technologies Inc., Beijing, China), in accordance with the manufacturer’s instructions [37].

### 4.6. ELISA Assay

The cortex and hippocampus tissues of mice were homogenized in cool saline and centrifugated at 4500 rpm for 10 min at 4 °C. The concentration of protein in supernatant was quantified by the BCA (Thermo, Waltham, MA, USA) method. The levels of inflammatory factors TNF-a, IL-1β, IL-6, MCP-1 and ICAM-1 into cell culture supernatant or tissue supernatant were detected by ELISA kits in accordance with the manufacturer’s instructions [37].

### 4.7. Total RNA Extraction and Real-Time PCR Analysis

The BV2 cells were seeded in 6-well plates at 1 × 10^6^ cells/well. After ST2825 treatment for 12 h and LPS stimulation for 6 h, RNA was extracted using TRIZOL reagent (Ambion, Carlsbad, CA, USA), in accordance with the manufacturer’s protocol. For mRNA expression analysis, cDNA was synthesized from 1 µg total RNA using MonScriptTMR-TIII All-in-One Mix (Monad Biotech Co., Ltd., Wuhan, China) in accordance with the manufacturer’s protocol. A 2uL cDNA template was used for real-time PCR with 〖SYBR〗^®^ qPCR Master Mix (Vazyme Biotech Co., Ltd., Nanjing, China). PCR was carried out as follows: 95 °C, initial denaturation of 30 s, followed by 39 cycles of 95 °C for 5 s, 60 °C for 30 s and 65 °C for 5 s. Consumables and reagents used for RNA extraction and PCR were all RNase-free. According to the relative quantification of 2^−^^ΔΔC_t^ method, the transcription levels of the target gene could be determined using β-actin as an internal reference (Liu et al., 2021). The primers used in the real-time PCR reactions included β-actin (forward, AGGCCAACCGTGAAAAGATG; reverse, TGGCGTGAGGGAGAGCATAG) [7], TNF-α (forward, CCACGCTCTTCTGTCTACTG; reverse, ACTTGGTGGTTTGCTACGAC) [38], IL-1β (forward, CCAGGATGAGGACATGAGCA; reverse, CGGAGCCTGTAGTGCAGTTG) [7], IL-6 (forward, AAAGAGTTGTGCAATGGCAATTCT; reverse, AAGTGCATCATCGTTGTTCATACA) [18], MCP-1 (forward, CCCAATGAGTAGGCTGGAGA; reverse, AAAATGGATCCACACCTTGC) [39], iNOS (forward, ACAGGGAGAAAGCGCAAAAC; reverse, TGTGGCCTTGTGGTGAAGAG) [40], COX-2 (forward, AACCGAGTCGTTCTGCCAAT; reverse, CTAGGGAGGGGACTGCTCAT) [41].

### 4.8. Western Blot Analysis

Total proteins of whole cell lysates from BV2 cells were obtained by Radio Immunoprecipitation Assay (RIPA) containing cocktail protease inhibitors (Thermo, Waltham, MA, USA). Protein concentration was determined using a BCA assay kit. An equal amount of protein from each sample was resolved using 10% sodium dodecyl sulfate-polyacrylamide gel electrophoresis (SDS-PAGE) and transferred to polyvinylidene fluoride (PVDF) membrane. The membranes were blocked with 5% skimmed milk, sequentially incubated with the primary antibody (iNOS, 1:1000; COX-2, 1:1000; phospho-NF-κB p65, 1:1000; NF-κB p65, 1:1000; phospho-IκBα, 1:1000; IκBα, 1:1000; NLRP3, 1:1000; cleaved caspase-1, 1:1000; IL-1β, 1:1000; IL-18, 1:1000; β-actin, 1:1000) overnight at 4 ℃, and with the horseradish peroxidase-conjugated secondary antibody for 2 h at room temperature and, finally, detected by enhanced ECL system after washing. Intensities of band signals were measured by Gel-Pro software (Molecular Imager ChemiDoc XRS+System, Bio-Rad, Irvine, CA, USA), and standardized by the value of β-actin as an internal reference except for phospho-NF-κB and phospho-IκBα [7].

### 4.9. Intracellular Reactive Oxygen Species (ROS) Assay

After 24 h of LPS stimulation, the BV2 cells in the 6-well plates were collected and stained with 5 μM DCFH-DA (Beyotime Institute of Biotechnology, Haimen, China) in the dark for 30 min at 37 ℃. After washing twice with PBS, 10,000 viable BV2 cells were immediately analyzed in a flow cytometer (BD Biosciences, San Jose, CA, USA) with an excitation wavelength of 488 nm and an emission wavelength of 525 nm [42].

### 4.10. Statistical Analysis

The data obtained from each mouse were included in the statistics. All data were analyzed with GraphPad Prism 7.0 software (GraphPad Software, Inc., La Jolla, CA, USA) using one-way ANOVA and Dunnett’s multiple comparisons test, and *p* < 0.05 was taken to be significant. Only the experimenters performing the experimental intervention knew the allocations of the experimental group. The data analysts remained blinded to the experimental group allocations until all analyses were completed.

## 5. Conclusions

ST2825 suppressed the LPS-induced inflammation in BV2 microglia cells via decreasing the release of inflammatory factors, inactivating NF-κB and the ROS/NLRP3/cleaved caspase-1 signaling pathway. This study suggests MyD88 as a potential therapeutic target, and ST2825 as a potential therapeutic agent for neuroinflammation in CNS disease. However, further research is needed to determine which immune pathways or molecules involved in neuroinflammation should be targeted.

## Data Availability

The data presented in this study are available on request from the corresponding author.

## Sample Availability

Samples of the compounds are not available from the authors.

## References

[1] DiSabato D.J., Quan N., Godbout J.P. (2016). Neuroinflammation: The devil is in the details. J. Neurochem..

[2] Lim H.S., Kim Y.J., Kim B.Y., Park G., Jeong S.J. (2018). The Anti-neuroinflammatory Activity of Tectorigenin Pretreatment via Downregulated NF-κB and ERK/JNK Pathways in BV-2 Microglial and Microglia Inactivation in Mice With Lipopolysaccharide. Front. Pharmacol..

[3] Nguyen P.L., Bui B.P., Lee H., Cho J. (2021). A Novel 1,8-Naphthyridine-2-Carboxamide Derivative Attenuates Inflammatory Responses and Cell Migration in LPS-Treated BV2 Cells via the Suppression of ROS Generation and TLR4/Myd88/NF-κB Signaling Pathway. Int. J. Mol. Sci..

[4] Hickman S., Izzy S., Sen P., Morsett L., El Khoury J. (2018). Microglia in neurodegeneration. Nat. Neurosci..

[5] Chen T., Luo W., Wu G., Wu L., Huang S., Li J., Wang J., Hu X., Huang W., Liang G. (2019). A novel MyD88 inhibitor LM9 prevents atherosclerosis by regulating inflammatory responses and oxidative stress in macrophages. Toxicol. Appl. Pharmacol..

[6] Ramírez-Pérez S., Hernández-Palma L.A., Oregon-Romero E., Anaya-Macías B.U., García-Arellano S., González-Estevez G., Muñoz-Valle J.F. (2020). Downregulation of Inflammatory Cytokine Release from IL-1β and LPS-Stimulated PBMC Orchestrated by ST2825, a MyD88 Dimerisation Inhibitor. Molecules.

[7] Liu M., Zhang S.S., Liu D.N., Yang Y.L., Wang Y.H., Du G.H. (2021). Chrysomycin A Attenuates Neuroinflammation by Down-Regulating NLRP3/Cleaved Caspase-1 Signaling Pathway in LPS-Stimulated Mice and BV2 Cells. Int. J. Mol. Sci..

[8] Batista C.R.A., Gomes G.F., Candelario-Jalil E., Fiebich B.L., De Oliveira A.C.P. (2019). Lipopolysaccharide-Induced Neuroinflammation as a Bridge to Understand Neurodegeneration. Int. J. Mol. Sci..

[9] Garces K., Carmy T., Illiano P., Brambilla R., Hackam A.S. (2020). Increased Neuroprotective Microglia and Photoreceptor Survival in the Retina from a Peptide Inhibitor of Myeloid Differentiation Factor 88 (MyD88). J. Mol. Neurosci..

[10] Long T., Liu Z., Shang J., Zhou X., Yu S., Tian H., Bao Y. (2018). Polygonatum sibiricum polysaccharides play anti-cancer effect through TLR4-MAPK/NF-κB signaling pathways. Int. J. Biol. Macromol..

[11] Subhramanyam C.S., Wang C., Hu Q., Dheen S.T. (2019). Microglia-mediated neuroinflammation in neurodegenerative diseases. Semin. Cell Dev. Biol..

[12] Pierre W.C., Smith P., Londono I., Chemtob S., Mallard C., Lodygensky G.A. (2017). Neonatal microglia: The cornerstone of brain fate. Brain Behav. Immun..

[13] Ko W., Sohn J.H., Jang J.H., Ahn J.S., Kang D.G., Lee H.S., Kim J.S., Kim Y.C., Oh H. (2016). Inhibitory effects of alternaramide on inflammatory mediator expression through TLR4-MyD88-mediated inhibition of NF-kB and MAPK pathway signaling in lipopolysaccharide-stimulated RAW264.7 and BV2 cells. Chem. Biol. Interact..

[14] Alfonso-Loeches S., Pascual-Lucas M., Blanco A.M., Sanchez-Vera I., Guerri C. (2010). Pivotal role of TLR4 receptors in alcohol-induced neuroinflammation and brain damage. J. Neurosci..

[15] Qi M., Yin L., Xu L., Tao X., Qi Y., Han X., Wang C., Xu Y., Sun H., Liu K. (2016). Dioscin alleviates lipopolysaccharide-induced inflammatory kidney injury via the microRNA let-7i/TLR4/MyD88 signaling pathway. Pharmacol. Res..

[16] LaRosa D.F., Rahman A.H., Turka L.A. (2007). The innate immune system in allograft rejection and tolerance. J. Immunol..

[17] Saikh K.U., Morazzani E.M., Piper A.E., Bakken R.R., Glass P.J. (2020). A small molecule inhibitor of MyD88 exhibits broad spectrum antiviral activity by up regulation of type I interferon. Antivir. Res..

[18] Chourbaji S., Urani A., Inta I., Sanchis-Segura C., Brandwein C., Zink M., Schwaninger M., Gass P. (2006). IL-6 knockout mice exhibit resistance to stress-induced development of depression-like behaviors. Neurobiol. Dis..

[19] Chen J., Ullah H., Zheng Z., Gu X., Su C., Xiao L., Wu X., Xiong F., Li Q., Zha L. (2020). Soyasaponins reduce inflammation by downregulating MyD88 expression and suppressing the recruitments of TLR4 and MyD88 into lipid rafts. BMC Complementary Med. Ther..

[20] Wang N., Han X., Liu H., Zhao T., Li J., Feng Y., Mi X., Zhang Y., Chen Y., Wang X. (2017). Myeloid differentiation factor 88 is up-regulated in epileptic brain and contributes to experimental seizures in rats. Exp. Neurol..

[21] Xiang W., Chao Z.Y., Feng D.Y. (2015). Role of Toll-like receptor/MYD88 signaling in neurodegenerative diseases. Rev. Neurosci..

[22] Kawai T., Adachi O., Ogawa T., Takeda K., Akira S. (1999). Unresponsiveness of MyD88-deficient mice to endotoxin. Immunity.

[23] Miao Y., Ding Z.C., Zou Z.M., Yang Y., Yang M., Zhang X.Q., Li Z.Y., Zhou L., Zhang L.M., Zhang X. (2020). Inhibition of MyD88 by a novel inhibitor reverses two-thirds of the infarct area in myocardial ischemia and reperfusion injury. Am. J. Transl. Res..

[24] Singh A., Devkar R., Basu A. (2020). Myeloid Differentiation Primary Response 88-Cyclin D1 Signaling in Breast Cancer Cells Regulates Toll-Like Receptor 3-Mediated Cell Proliferation. Front. Oncol..

[25] Yan H., Zhang D., Wei Y., Ni H., Liang W., Zhang H., Hao S., Jin W., Li K., Hang C.H. (2017). Inhibition of myeloid differentiation primary response protein 88 provides neuroprotection in early brain injury following experimental subarachnoid hemorrhage. Sci. Rep..

[26] Ahmed H., Khan M.A., Kahlert U.D., Niemelä M., Hänggi D., Chaudhry S.R., Muhammad S. (2021). Role of Adaptor Protein Myeloid Differentiation 88 (MyD88) in Post-Subarachnoid Hemorrhage Inflammation: A Systematic Review. Int. J. Mol. Sci..

[27] Saikh K.U. (2021). MyD88 and beyond: A perspective on MyD88-targeted therapeutic approach for modulation of host immunity. Immunol. Res..

[28] Turner M.D., Nedjai B., Hurst T., Pennington D.J. (2014). Cytokines and chemokines: At the crossroads of cell signalling and inflammatory disease. Biochim. Biophys. Acta.

[29] Nam H.Y., Nam J.H., Yoon G., Lee J.Y., Nam Y., Kang H.J., Cho H.J., Kim J., Hoe H.S. (2018). Ibrutinib suppresses LPS-induced neuroinflammatory responses in BV2 microglial cells and wild-type mice. J. Neuroinflammation.

[30] Palomino D.C., Marti L.C. (2015). Chemokines and immunity. Einstein (Sao Paulo).

[31] Bui T.M., Wiesolek H.L., Sumagin R. (2020). ICAM-1: A master regulator of cellular responses in inflammation, injury resolution, and tumorigenesis. J. Leukoc. Biol..

[32] Matsuo K., Yabuki Y., Fukunaga K. (2017). Combined l-citrulline and glutathione administration prevents neuronal cell death following transient brain ischemia. Brain Res..

[33] Baek H.S., Min H.J., Hong V.S., Kwon T.K., Park J.W., Lee J., Kim S. (2020). Anti-Inflammatory Effects of the Novel PIM Kinase Inhibitor KMU-470 in RAW 264.7 Cells through the TLR4-NF-κB-NLRP3 Pathway. Int. J. Mol. Sci..

[34] Park J., Min J.S., Kim B., Chae U.B., Yun J.W., Choi M.S., Kong I.K., Chang K.T., Lee D.S. (2015). Mitochondrial ROS govern the LPS-induced pro-inflammatory response in microglia cells by regulating MAPK and NF-κB pathways. Neurosci. Lett..

[35] Cheng X., Yang Y.L., Yang H., Wang Y.H., Du G.H. (2018). Kaempferol alleviates LPS-induced neuroinflammation and BBB dysfunction in mice via inhibiting HMGB1 release and down-regulating TLR4/MyD88 pathway. Int. Immunopharmacol..

[36] Liu M., Yang Y.L., Zhang S.S., Liu D.N., Fang L.H., Du G.H., Wang Y.H. (2021). RKC-B1 Blocks Activation of NF-κB and NLRP3 Signaling Pathways to Suppress Neuroinflammation in LPS-Stimulated Mice. Mar. Drugs.

[37] Yang H., Cheng X., Yang Y.L., Wang Y.H., Du G.H. (2017). Ramulus Cinnamomi extract attenuates neuroinflammatory responses via downregulating TLR4/MyD88 signaling pathway in BV2 cells. Neural Regen. Res..

[38] Chen Y.F., Wang K., Zhang Y.Z., Zheng Y.F., Hu F.L. (2016). In Vitro Anti-Inflammatory Effects of Three Fatty Acids from Royal Jelly. Mediat. Inflamm..

[39] Qian B.Z., Li J., Zhang H., Kitamura T., Zhang J., Campion L.R., Kaiser E.A., Snyder L.A., Pollard J.W. (2011). CCL2 recruits inflammatory monocytes to facilitate breast-tumour metastasis. Nature.

[40] Ataie Z., Dastjerdi M., Farrokhfall K., Ghiravani Z. (2021). The Effect of Cinnamaldehyde on iNOS Activity and NO-Induced Islet Insulin Secretion in High-Fat-Diet Rats. Evid. Based Complementary Altern. Med..

[41] Xu X., Xu H., Ren F., Huang L., Xu J., Li F. (2021). Protective effect of scorpion venom heat-resistant synthetic peptide against PM2.5-induced microglial polarization via TLR4-mediated autophagy activating PI3K/AKT/NF-κB signaling pathway. J. Neuroimmunol..

[42] Kim S.Y., Jin C.Y., Kim C.H., Yoo Y.H., Choi S.H., Kim G.Y., Yoon H.M., Park H.T., Choi Y.H. (2019). Isorhamnetin alleviates lipopolysaccharide-induced inflammatory responses in BV2 microglia by inactivating NF-κB, blocking the TLR4 pathway and reducing ROS generation. Int. J. Mol. Med..

